# Incidence and impact of sepsis on long-term outcomes after subarachnoid hemorrhage: a prospective observational study

**DOI:** 10.1186/s13613-019-0562-3

**Published:** 2019-08-20

**Authors:** Bruno Gonçalves, Pedro Kurtz, Ricardo Turon, Thayana Santos, Marco Prazeres, Cassia Righy, Fernando Augusto Bozza

**Affiliations:** 1 Instituto Estadual do Cérebro Paulo Niemeyer, Rua do Rezende, 156, Centro, Rio de Janeiro, RJ 20230-026 Brazil; 2 Laboratório de Medicina Intensiva, Instituto Nacional de Infectologia Evandro Chagas (INI), Fundação Oswaldo Cruz (FIOCRUZ), Av. Brasil, 4365, Manguinhos, Rio de Janeiro, RJ Zip code: 21045-900 Brazil; 3 D’Or Institute for Research and Education (IDOR), R. Diniz Cordeiro 30, Rio de Janeiro, Brazil

**Keywords:** Subarachnoid hemorrhage, Sepsis, Infection, Critical care outcomes

## Abstract

**Background:**

Aneurysmal subarachnoid hemorrhage (SAH) is an acute cerebrovascular disease associated with high mortality and long-term functional impairment among survivors. Systemic inflammatory responses after acute injury and nosocomial infections are frequent complications, making the management of these patients challenging. Here, we hypothesized that sepsis might be associated with early and long-term mortality and functional outcomes. Our objective was to define the incidence of sepsis, diagnosed prospectively with the Sepsis-3 criteria, and to determine its impact on mortality and functional outcomes of patients with SAH.

**Methods:**

We prospectively included all adult patients with aneurysmal SAH admitted to the intensive care unit (ICU) of a reference center between April 2016 and May 2018. Daily clinical and laboratory follow-up data were analyzed during the first 14 days, with data collected on sepsis according to the Sepsis-3 criteria. The main outcome was the functional outcome using the Modified Rankin Scale (mRS), which was assessed at hospital discharge and 3, 6 and 12 months post-discharge.

**Results:**

In total, 149 patients were enrolled. The incidence of sepsis was 28%. Multivariable logistic regression analysis revealed that death or functional dependence (defined as an mRS score of 4 to 6) at hospital discharge was independently associated with sepsis (OR 3.4, 95% CI 1.16–9.96, *p* = 0.026) even after controlling for World Federation of Neurological Surgeons (WFNS) Scale (OR 4.66, 95% CI 1.69–12.88, *p* = 0.003), hydrocephalus (OR 4.55, 95% CI 1.61–12.85, *p* = 0.004) and DCI (OR 3.86, 95% CI 1.39–10.74, *p* = 0.01). Long-term follow-up mortality rates were significantly different in the septic and nonseptic groups (log-rank test *p* < 0.0001). The mortality rate of septic patients was 52.5%, and that of nonseptic patients was 16%.

**Conclusion:**

Sepsis plays a significant role in the outcomes of patients with SAH, affecting both mortality and long-term functional outcomes. Combining high-level neurocritical care management of neurological complications and the optimal diagnosis and management of sepsis may effectively reduce secondary brain injury and improve patients’ outcomes after SAH.

## Background

Aneurysmal subarachnoid hemorrhage (SAH) is an acute cerebrovascular disease with devastating consequences, including high mortality and long-term functional impairment among survivors [1]. Several brain-specific mechanisms of injuries secondary to SAH have been associated with worse functional outcomes, including early injuries, vasospasms and delayed cerebral ischemia (DCI), neuroinflammation or impaired cerebral autoregulation [2–6]. However, there are few options for treating or preventing such complications of SAH. On the other hand, systemic (and potentially preventable) complications such as nosocomial infections, sepsis and organ dysfunction may also alter the course of the disease and worsen survival as well as functional capacity [7]. The incidence of systemic inflammatory response syndrome (SIRS) after SAH may be up to 83% of patients, and the incidence of sepsis varies between 10 and 20%, with a strong association with poor outcomes [8, 9].

Approximately 30% of patients with SAH will develop nosocomial infection during the course of hospitalization [10]. However, most of the available data are derived from retrospective studies or cohort studies with imprecise definitions of infections or sepsis. The diagnosis of sepsis is challenging in patients with SAH due to the high incidence of early SIRS and the lack of reliable biomarkers that can be used to differentiate SIRS and sepsis [8, 9]. Therefore, there is a risk of sepsis misdiagnosis, which can potentially lead to the under- or overtreatment of SAH patients. Considering the previously exposed, the objective of this investigation was to define the incidence of sepsis, diagnosed prospectively with the Sepsis-3 criteria, and its impact on mortality and functional outcomes of patients with SAH.

## Methods

### Design and setting

We prospectively included all adult patients (≥ 18 years) admitted to the neurological intensive care unit (ICU) of the Paulo Niemeyer State Brain Institute (Rio de Janeiro, Brazil) with aneurysmal SAH between April 2016 and May 2018. The Institute is a reference center for neurovascular diseases and receives approximately 70–100 SAH patients per year from a statewide public healthcare network in Brazil.

The present study was approved by the Ethics Review Board of the Evandro Chagas National Institute of Infectious Diseases—Oswaldo Cruz Foundation (Rio de Janeiro). The data were deidentified by assigning each patient a unique identification number. SAH was diagnosed by findings from the initial computed tomography (CT) scan or by xanthochromia in the cerebrospinal fluid if the findings from the CT scan were normal. Patients who were admitted 30 days or more after a hemorrhagic ictus were excluded. We also excluded patients who were pregnant or had a life expectancy of less than 48 h after admission to the ICU.

### Clinical assessment

Demographic data, social and medical history, and clinical features at onset were obtained shortly after admission. Neurological status was assessed with the Glasgow Coma Scale (GCS) [11] and the World Federation of Neurological Surgeons (WFNS) Scale [12]. Systemic disease severity was assessed with the Acute Physiology and Chronic Health Evaluation II (APACHE II) score [13]. Admission and follow-up computed tomography (CT) scans during hospitalization were evaluated by using the modified Fisher scale [14] and for the presence of global cerebral edema and infarction. Daily clinical and laboratory follow-ups were analyzed during the first 14 days of hospitalization or up to the ICU discharge. Data were collected daily during the hospital stay by two investigators (TS and MP), using an electronic case report form (through Research Electronic Data Capture—REDCap). For all patients, the following daily evaluations were also performed: the presence of SIRS and the presence of sepsis or septic shock (according to the Sepsis-3 criteria) [7]; organ dysfunction using the sequential organ failure assessment (SOFA) [15]; and the presence of infection according to clinical and the Centers for Disease Control and Prevention (CDC) criteria. The clinical variables and the diagnosis of infection and sepsis were validated both by the research study team (BG, RT, PK and CR) and through adjudication by an independent infectious disease specialist.

### Outcome assessment

The main outcome was the functional outcome, using the Modified Rankin Scale (mRS) score [16]. Scores were prospectively assessed at hospital discharge and 3, 6 and 12 months post-discharge. Long-term outcomes (at 3, 6 and 12 months) were assessed via a telephone interview with the validated mRS score [17]. The interviews were conducted three times during the course of the study; thus, patients included in the final months of the study only had the 3- or 6-month follow-up. Any patient without data available after hospital discharge was considered lost to follow-up. Functional outcome was dichotomized to a poor outcome (defined as mRS 4 to 6) and a good outcome (mRS 0 to 3). Hospital complications after SAH were diagnosed by the clinical team and adjudicated by the research study team (BG, RT, PK and CR) on a weekly basis. DCI was defined as an otherwise unexplained clinical deterioration (such as a new focal deficit, decrease in the level of consciousness or both) or a new infarct shown on the CT scan that was not visible in the admission or the immediate postoperative CT scan, or both, after the exclusion of other potential causes of clinical deterioration. Postoperative deterioration due to operative complications was defined as any neurological worsening or a new infarct within 48 h after the aneurysm repair procedure. Other complications, such as hydrocephalus (defined in this study as the need for cerebrospinal fluid drainage by external ventricular drain or lumbar puncture for that end), rebleeding (defined as a new brain hemorrhage on CT and not related to a surgical procedure), vasospasm (defined as arterial narrowing on cerebral angiogram or mean velocity higher than 120 cm/s and Lindegaard index higher than 3 on transcranial Doppler) and seizures, were also recorded. Aneurysm rebleeding was defined as an acute neurological deterioration with a new hemorrhage apparent on the CT scan.

### Statistical analysis

Data are presented as means (standard deviations) or medians (interquartile ranges) for continuous variables and as absolute numbers and percentages for categorical variables. Univariate associations were tested by using the Chi-square or Fisher’s exact test for categorical variables, the two-tailed *t* test for normally distributed continuous variables and the Mann–Whitney *U* test for nonnormally distributed continuous variables. Logistic regression was used to test the association between sepsis and mortality and functional outcome using known predictors of poor outcome as covariates. An initial Multivariable analysis was performed to determine the relationship between premorbid demographic, admission clinical and radiographic variables, as well as complications and surgical treatment offered, with functional outcome at hospital discharge. We initially considered for the multivariable model variables associated with poor outcome with a *p* value lower than 0.1 in univariate analysis. Adjusted odds ratios for specific hospital complications, including sepsis, were calculated by sequentially adding each of these factors to the baseline model to evaluate their unique contribution. A Hosmer–Lemeshow test was used to test the goodness of fit of each model. The final model included age, sex and variables that remained significantly associated with the outcome variable. Potential multicollinearity between the parameters of the final regression model was assessed by calculating tolerance and variance inflation factor coefficients. A Kaplan–Meier survival curve was derived from the log-rank test for the septic and nonseptic groups. The significance was set to 0.05 for all analyses. All analyses were performed with commercially available statistical software (SPSS version 19.0; SPSS Inc., now part of IBM Corporation, Armonk, NY, USA).

## Results

### Baseline characteristics

In total, 149 patients were enrolled in the study. There were no patients admitted to the ICU who met the exclusion criteria during the period of the study. Age ranged from 22 to 79 years (median 55 years), and most patients were female (109–73%). The demographic and baseline characteristics of all patients are detailed in Table 1. In-hospital mortality was 17% (25 patients), and 69 patients (46%) had poor outcomes at hospital discharge (mRS of 4–6).

**Table 1:** Patient demographics and baseline characteristics

*N* = 149	*N* (%)
Median age (range)	55 (22–79)
Female	109 (73%)
Arterial hypertension	100 (67%)
Smoking	44 (30%)
Alcoholism	20 (13%)
Diabetes mellitus	12 (8%)
WFNS grade (I–V)	
I	63 (42%)
II	32 (21%)
III	7 (5%)
IV	29 (19%)
V	18 (12%)
Modified Fisher (0–4)	
0	6 (4%)
1	14 (9%)
2	16 (11%)
3	65 (44%)
4	48 (32%)
Mechanical ventilation	50 (34%)
SIRS	122 (82%)
Sepsis	41 (28%)
Hydrocephalus	45 (30%)
Rebleeding	9 (6%)
Vasospasm	55 (37%)
Postoperative neurological deterioration	47 (32%)
DCI	47 (32%)
Hospital mortality	25 (17%)
Poor outcome at discharge (Modified Rankin 4–6)	69 (46%)

### Incidence of sepsis and SIRS

Fifty-six patients (38%) developed 60 infectious events (four patients had two distinct episodes of infection). The events were classified as follows: fourteen episodes of ventilator-associated pneumonias (VAP), thirteen nosocomial pneumonias, eleven tracheobronchitis, nine meningitis/ventriculitis, six urinary tract infections, two bloodstream infections, one infected pressure ulcer, one dental abscess and three indeterminate infections. Out of all episodes of infection, 27 (45%) were classified as pneumonias. The incidence of sepsis was 28% (41/149 patients), and among those, 18 patients developed septic shock (12% of all patients, 44% of the septic patients). Among septic patients, the initial infection was VAP in nine patients, nosocomial pneumonia in nine, tracheobronchitis in eight, meningitis/ventriculitis in three, urinary tract infections in six, bloodstream infections in two, infected pressure ulcer in one and infections of indeterminate origin in three patients. SIRS was present in 122 patients (82%). The median time between admission and the diagnosis of sepsis was 3 days (interval of 1–14 days). The characteristics of each group, septic and nonseptic, are detailed in Table 2.

**Table 2:** Baseline characteristics of septic and nonseptic groups (*N*/percentage or range/mean)

	Septic	Nonseptic	*p* value
	*N* = 41	*N* = 108	
Age	23–77 (54)	22–79 (52)	0.4
Female gender	27 (66%)	82 (76%)	0.22
Arterial hypertension	34 (83%)	66 (61%)	0.01
Diabetes mellitus	5 (12%)	7 (6%)	0.31
Alcoholism	7 (17%)	13 (12%)	0.42
Smoking	12 (29%)	32 (29%)	1
APACHE II	5–36 (16.22)	2–31 (10.81)	< 0.00001
Cardiovascular SOFA at admission	0–4 (0.9)	0–4 (0.26)	0.0005
GCS at admission	3–15 (9.51)	3–15 (13.44)	< 0.00001
WFNS 4–5	25 (61%)	22 (20%)	< 0.0001
Modified Fisher 3–4	34 (83%)	79 (73%)	0.28
Mechanical ventilation	31 (76%)	19 (17%)	< 0.00001
Hydrocephalus	23 (56%)	22 (20%)	< 0.0001
Rebleeding	1 (2%)	8 (7%)	0.45
Endovascular treatment	22 (54%)	28 (26%)	0.002
Surgical treatment	17 (41%)	75 (69%)	0.002

### Outcomes at hospital discharge

**Fig. 1:**
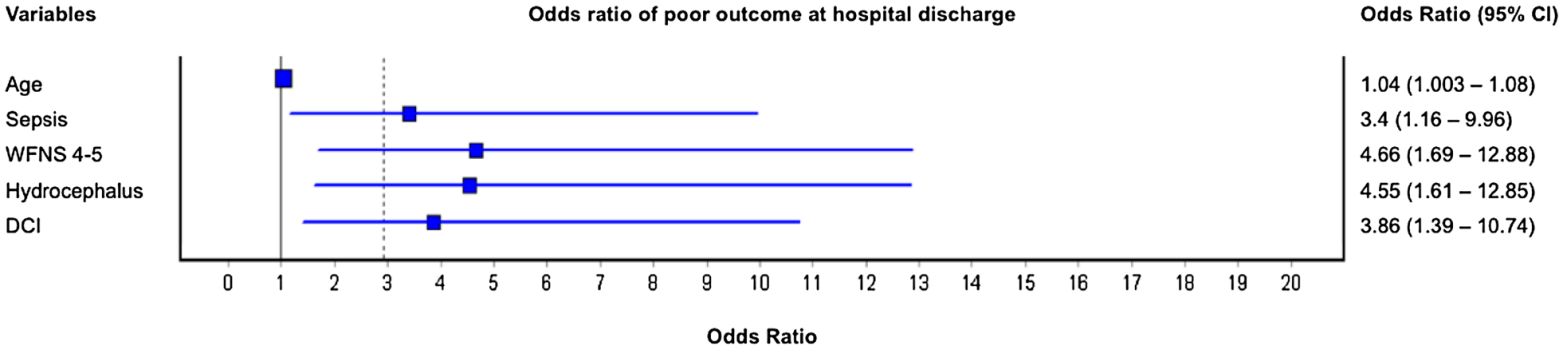
Risk factors for poor outcome at discharge—multivariate analysis

In the univariate analysis, age over 55 years (*p* = 0.049), sepsis (*p* < 0.0001), poor grade SAH (WFNS scale of 4–5) (*p* < 0.0001), modified Fisher scale of 3–4 (*p* = 0.01), hydrocephalus (*p* < 0.0001), vasospasm (*p* = 0.004), postoperative neurological deterioration (*p* = 0.027) and DCI (*p* < 0.0001) were associated with a poor outcome. Multivariable logistic regression analysis, with outcomes at hospital discharge, revealed that death or functional dependence (mRS of 4 to 6) at hospital discharge were independently associated with sepsis (OR 3.4, 95% CI 1.16–9.96, *p* = 0.026) even after controlling for WFNS (OR 4.66, 95% CI 1.69–12.88, *p* = 0.003), hydrocephalus (OR 4.55, 95% CI 1.61–12.85, *p* = 0.004) and DCI (OR 3.86, 95% CI 1.39–10.74, *p* = 0.01) (Table 3). SIRS, however, was not independently associated with a poor functional outcome. The data of the multivariable analysis are presented in Fig. 1.

**Table 3:** Study variables and association with functional outcome (at discharge)

	Unfavorable outcome	Favorable outcome	Univariate analysis	Multivariable analysis	
	(Modified Rankin 4–6) *N* = 69	(Modified Rankin 0–3) *N *= 80	*p* value	OR (95% CI)	*p* value
Age	57 (45.5–62)	52 (44–58)	0.049	1.04 (1.003–1.08)	0.033
Female gender	51 (73.9%)	58 (72.5%)	0.846		
Infection	42 (60.9%)	14 (17.5%)	< 0.0001		
Pneumonia^a^	23 (33.3%)	4 (5%)	< 0.0001		
SIRS	65 (94.2%)	57 (71.2%)	0.0006		
Sepsis	34 (49.3%)	7 (8.8%)	< 0.0001	3.4 (1.16–9.96)	0.026
WFNS 4–5	38 (55.1%)	9 (11.3%)	< 0.0001	4.66 (1.69–12.88)	0.003
Modified Fisher 3–4	59 (85.5%)	54 (67.5%)	0.01		
Hydrocephalus	36 (52.2%)	9 (11.3%)	< 0.0001	4.55 (1.61–12.85)	0.004
Rebleeding	4 (5.8%)	5 (6.3%)	1		
Vasospasm	34 (49.3%)	21 (26.3%)	0.004		
Post-op deterioration	28 (40.6%)	19 (23.8%)	0.027		
DCI	38 (55.1%)	9 (11.3%)	< 0.0001	3.86 (1.39–10.74)	0.01

^a^Both ventilator-associated pneumonia and nosocomial pneumonia

### Long-term outcomes

Follow-up data were acquired for 136 patients (13 patients were lost to follow-up—9%, 12 of those patients were in the nonseptic group). Data were available for 95 patients at 12 months (70%), 28 patients at 6 months (20.5%) and 13 patients at 3 months (9.5%). The long-term mortality rate (using the last follow-up available for each patient until 12 months) was 26% (36 out of 136 patients), and the poor outcome rate was 33% (46 out of 136 patients).

**Fig. 2:**
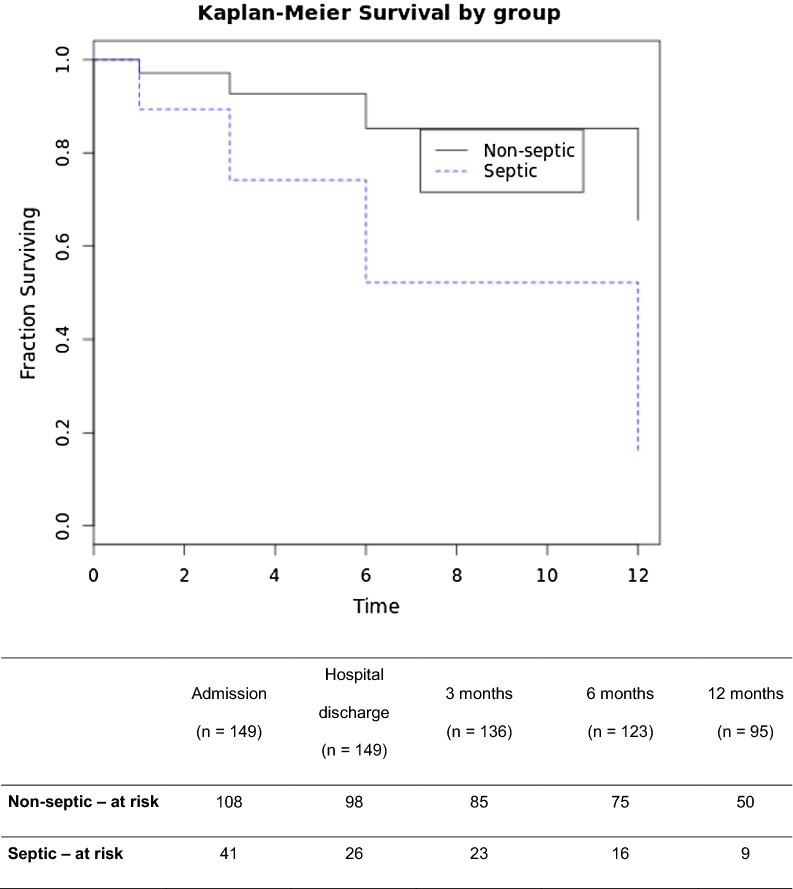
Kaplan–Meier survival curves of septic × nonseptic patients with SAH. Log-rank test *p* < 0.0001. Time to death or last follow-up in months

**Fig. 3:**
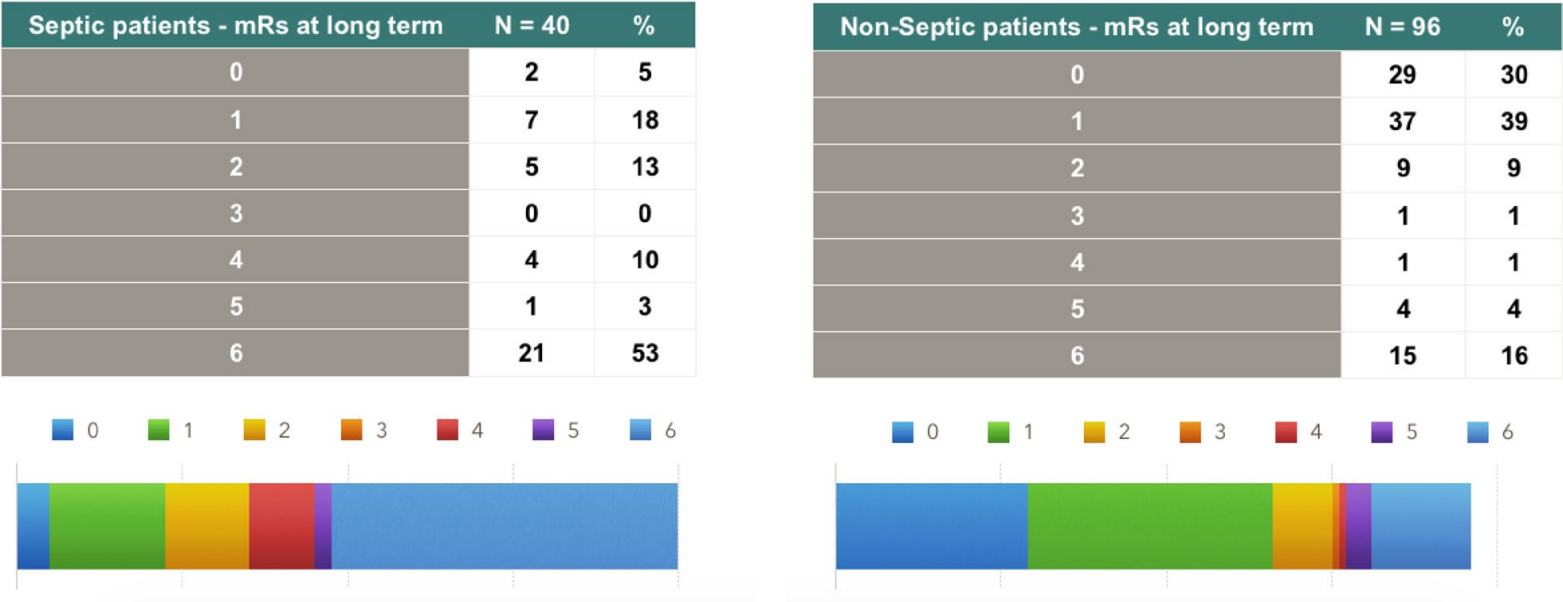
Long-term outcomes on septic and nonseptic patients (poor outcome—mRS 4 to 6 *p* < 0.0001)

Mortality rates and functional outcomes for the long-term follow-up were significantly different in the septic and nonseptic groups, with higher rates in septic patients (log-rank test *p* < 0.0001). The long-term mortality rate of septic patients was 52.5% (21 out of 40 patients), and that of nonseptic patients was 16% (15 out of 96). Long-term poor outcome rates were 66% (26 out of 40) for the septic group and 21% (20 out of 96) for nonseptic patients. The Kaplan–Meier survival curve is depicted in Fig. 2, and Fig. 3 shows the long-term outcomes, divided by the mRS, in both the septic and nonseptic groups.

## Discussion

In this study, we present the results of a prospective cohort study of SAH patients with functional outcomes (measured by the mRS score) and mortality collected during a maximum of 12 months of follow-up. We prospectively investigated the incidence of sepsis during the first 14 days of the hospital stay, with an incidence of 28%. Sepsis was independently associated with a threefold increase in poor functional outcomes and long-term (using the last data point for each patient) mortality (52.5% for septic patients and 16% for nonseptic patients—log-rank test *p* < 0.0001).

Aneurysmal SAH is a severe cerebrovascular event that may lead to lifelong disabilities. Mortality can be as high as 15% prior to hospital admission and may reach 40 to 45% in the 30 days after the bleed [3, 18]. After the initial ictus, a number of complications leading to secondary brain injury can further impact outcomes. Sepsis, a complex syndrome where a dysregulated inflammatory response to an infection leads to organ dysfunction, is one of these complications [7].

Regardless of adjustment, in the multivariable analysis, for known neurological factors that affect outcomes (poor grade, hydrocephalus and DCI), sepsis was still independently associated with an unfavorable outcome in our cohort. Some of these neurological factors, such as poor clinical presentation, depend on the initial brain injury—such as a loss of consciousness, seizures and the bleed volume [14]. Others, such as DCI, remain poorly understood and have limited therapeutic options. Sepsis, on the other hand, may be prevented through measures that effectively reduce nosocomial infections. Moreover, accurate and early diagnosis of sepsis combined with an aggressive treatment for organ dysfunction may potentially improve outcomes for SAH patients [3, 19].

Although some studies have shown an association between sepsis and unfavorable outcomes after SAH [20–23], none used the current definition of sepsis (Sepsis-3.0). The previous definitions, which included the SIRS criteria, were less specific and did not clearly discriminate sepsis from a systemic inflammatory response due to noninfectious causes, such as an SAH. Two retrospective studies [24, 25] found associations between the SIRS burden and worse outcomes in SAH, with SIRS incidence ranging from 69 to 85%. Although present in 82% of our patients, SIRS was not associated with a poor outcome after adjusting for confounders. SIRS could be related to the severity of SAH presentation. Sepsis, on the other hand, could play a role in brain dysfunction developed after SAH, working as a second attack on a vulnerable brain. This phenomenon has been shown in other populations where sepsis leads to brain dysfunction, cognitive impairment, muscular loss and exacerbated end-organ dysfunction [19, 26].

The incidence of sepsis is higher than previously reported [8, 9]. The incidence of infection was also higher than previous reports (38%), with 45% of those infections presenting as pneumonia. This study is the first large cohort study of SAH patients with a sepsis diagnosis from low- and middle-income countries, which may explain the differences from the current literature, which mostly comprises European and North American data. Recently, a multicenter study from Brazil [27] has evaluated the national prevalence of sepsis on a single day and reported an incidence of 16.7% and a prevalence of 25% (meaning 30% of patients were septic on the day of the study). The in-hospital mortality rate was 56%. In contrast, the PRISM meta-analysis [28], which reported data from the USA, the UK and Australasia, showed a sepsis-related mortality rate of 25%. Again, data on sepsis, with the use of new criteria, is scarce in low- and middle-income countries and almost nonexistent in the neurocritical population, which may limit such comparisons.

Our study has limitations. First, our institution is a reference center, and the majority of patients are admitted 24 h after ictus. The delay in transfer may introduce selection bias, as patients with a very severe disease presentation, such as evidence of intracranial hypertension, may die before reaching our ICU. Also, that those patients who died early were excluded from our sample may be a reason for the overestimation in the incidence of sepsis in this cohort. Additionally, being a convenience sample, our cohort may be underpowered. Second, follow-up data were acquired for 136 patients (95 patients at 12 months, 28 patients at 6 months and 13 patients at 3 months), with 13 patients lost to follow-up. Third, sepsis diagnosis remains difficult in these patients, even with the new criteria. Confounders such as vasoactive drugs for induced hypertension, neurogenic pulmonary edema and stress-induced myocardial depression may simulate sepsis-induced organ dysfunction. In this population, it is even more difficult due to the neurological impairment due to the bleeding itself, which can elevate the SOFA score (specifically, the neurological SOFA) without sepsis, confounding the diagnosis. To avoid such bias, our sepsis diagnosis was validated by an external specialist (an infectious diseases’ specialist), and for the neurological SOFA, only the rise in the score was considered (not the baseline, if already altered) and when not due to SAH complications (such as DCI or hydrocephalus for instance). Finally, we do not have data on all of the patients’ brain images after discharge, so we cannot explore the hypothesis of sepsis worsening brain damage in more detail—however, this was already addressed by other authors [26] in previous studies.

Though the data indicate a relationship between sepsis and worse outcomes in this cohort, it is still scarce to define a cause–effect association with certainty, especially since the septic group was clearly more severe than the nonseptic group on presentation. However, the multivariate analysis still shows sepsis as an independent variable of worse outcomes. Thus, our study in fact serves to formulate and empower this hypothesis—further studies, with larger cohorts, should be analyzed in order to better clarify the matter.

That aside, a number of potential opportunities arise from a better understanding of the impact of sepsis in patients with SAH. Future studies should aim to develop novel and more accurate methods to improve sepsis diagnosis, such as early molecular detection of bacteria in the blood or monitoring of blood biomarkers (e.g., procalcitonin and C-reactive protein) [9, 10]. Additionally, strategies that aim to reduce early pneumonia, such as the preemptive administration of antibiotics in comatose patients, may potentially reduce the incidence of sepsis and benefit poor grade SAH patients [29].

Sepsis mortality has dropped in recent years in the general ICU population, mainly due to early recognition and effective treatment [30]. Our study shows an association between sepsis and outcomes in SAH; though it is underpowered to determine a cause–effect relationship, it still provides data to create this hypothesis. Based on our results, we believe that nosocomial infection prevention, improved diagnosis and optimal management of infection and sepsis in SAH patients may have a major impact on patients’ outcomes, especially in low- and middle-income countries.

## Conclusion

Sepsis can play a significant role in the outcomes of patients with SAH, affecting both mortality and long-term functional outcomes. Combining high-level neurocritical care management of neurological complications and optimal diagnosis and management of sepsis may effectively reduce secondary brain injury and improve patients’ outcomes after SAH.

## Data Availability

The datasets used and/or analyzed during the current study are available from the corresponding author upon reasonable request.

## Abbreviations

SAH: aneurysmal subarachnoid hemorrhage
DCI: delayed cerebral ischemia
SIRS: systemic inflammatory response syndrome
ICU: intensive care unit
CT: computed tomography
GCS: Glasgow Coma Scale
WFNS: World Federation of Neurological Surgeons Scale
APACHE II: Acute Physiology and Chronic Health Evaluation II
REDCap: Research Electronic Data Capture
SOFA: sequential organ failure assessment
CDC: Centers for Disease Control and Prevention
mRS: Modified Rankin Scale
VAP: ventilator-associated pneumonia

## References

[1] Okazaki T, Kuroda Y (2018). Aneurysmal subarachnoid hemorrhage: intensive care for improving neurological outcome. J Intensive Care..

[2] Nieuwkamp DJ, Setz LE, Algra A, Linn FHH, de Rooij NK, Rinkel GJE (2009). Changes in case fatality of aneurysmal subarachnoid haemorrhage over time, according to age, sex, and region: a meta-analysis. Lancet Neurol..

[3] Flynn L, Andrews P (2015). Advances in the understanding of delayed cerebral ischaemia after aneurysmal subarachnoid haemorrhage. F1000Res.

[4] Francoeur CL, Mayer SA (2016). Management of delayed cerebral ischemia after subarachnoid hemorrhage. Crit Care Lond Engl..

[5] Macdonald RL, Pluta RM, Zhang JH (2007). Cerebral vasospasm after subarachnoid hemorrhage: the emerging revolution. Nat Clin Pract Neurol..

[6] Gonçalves B, Turon R, Mendes A, Melo N, Lacerda P, Brasil P (2018). Effect of early brain infarction after subarachnoid hemorrhage: a systematic review and meta-analysis. World Neurosurg..

[7] Singer M, Deutschman CS, Seymour CW, Shankar-Hari M, Annane D, Bauer M (2016). The third international consensus definitions for sepsis and septic shock (Sepsis-3). JAMA.

[8] Festic E, Siegel J, Stritt M, Freeman WD (2014). The utility of serum procalcitonin in distinguishing systemic inflammatory response syndrome from infection after aneurysmal subarachnoid hemorrhage. Neurocrit Care.

[9] Oconnor E, Venkatesh B, Mashongonyika C, Lipman J, Hall J, Thomas P (2004). Serum procalcitonin and C-reactive protein as markers of sepsis and outcome in patients with neurotrauma and subarachnoid haemorrhage. Anaesth Intensive Care.

[10] Connolly ES, Rabinstein AA, Carhuapoma JR, Derdeyn CP, Dion J, Higashida RT (2012). Guidelines for the management of aneurysmal subarachnoid hemorrhage: a guideline for healthcare professionals from the American Heart Association/American Stroke Association. Stroke.

[11] Teasdale G, Jennett B (1976). Assessment and prognosis of coma after head injury. Acta Neurochir (Wien)..

[12] Report of World Federation Of Neurological Surgeons committee on a universal subarachnoid hemorrhage grading scale. J Neurosurg. 1988;68(6):985–6. 10.3171/jns.1988.68.6.0985 3131498

[13] Knaus WA, Draper EA, Wagner DP, Zimmerman JE (1985). APACHE II: a severity of disease classification system. Crit Care Med.

[14] Claassen J, Bernardini GL, Kreiter K, Bates J, Du YE, Copeland D (2001). Effect of cisternal and ventricular blood on risk of delayed cerebral ischemia after subarachnoid hemorrhage: the Fisher scale revisited. Stroke.

[15] Vincent JL, Moreno R, Takala J, Willatts S, De Mendonça A, Bruining H (1996). The SOFA (Sepsis-related Organ Failure Assessment) score to describe organ dysfunction/failure On behalf of the Working Group on Sepsis-Related Problems of the European Society of Intensive Care Medicine. Intensive Care Med.

[16] van Swieten JC, Koudstaal PJ, Visser MC, Schouten HJ, van Gijn J (1988). Interobserver agreement for the assessment of handicap in stroke patients. Stroke.

[17] Baggio JAO, Santos-Pontelli TEG, Cougo-Pinto PT, Camilo M, Silva NF, Antunes P (2014). Validation of a structured interview for telephone assessment of the modified Rankin Scale in Brazilian stroke patients. Cerebrovasc Dis Basel Switz..

[18] van Gijn J, Kerr RS, Rinkel GJE (2007). Subarachnoid haemorrhage. Lancet Lond Engl..

[19] Cecconi M, Evans L, Levy M, Rhodes A (2018). Sepsis and septic shock. Lancet Lond Engl..

[20] Abulhasan YB, Alabdulraheem N, Schiller I, Rachel SP, Dendukuri N, Angle MR (2018). Health care-associated infections after subarachnoid hemorrhage. World Neurosurg..

[21] Frontera JA, Fernandez A, Schmidt JM, Claassen J, Wartenberg KE, Badjatia N (2008). Impact of nosocomial infectious complications after subarachnoid hemorrhage. Neurosurgery..

[22] Taufique Z, May T, Meyers E, Falo C, Mayer SA, Agarwal S (2016). Predictors of poor quality of life 1 year after subarachnoid hemorrhage. Neurosurgery..

[23] Tseng M-Y, Hutchinson PJ, Kirkpatrick PJ (2010). Interaction of neurovascular protection of erythropoietin with age, sepsis, and statin therapy following aneurysmal subarachnoid hemorrhage. J Neurosurg.

[24] Dhar R, Diringer MN (2008). The burden of the systemic inflammatory response predicts vasospasm and outcome after subarachnoid hemorrhage. Neurocrit Care.

[25] Rass V, Gaasch M, Kofler M, Schiefecker AJ, Ianosi B-A, Rhomberg P (2018). Systemic inflammatory response syndrome as predictor of poor outcome in nontraumatic subarachnoid hemorrhage patients. Crit Care Med.

[26] Annane D, Sharshar T (2015). Cognitive decline after sepsis. Lancet Respir Med..

[27] Machado FR, Cavalcanti AB, Bozza FA, Ferreira EM, Angotti Carrara FS, Sousa JL (2017). The epidemiology of sepsis in Brazilian intensive care units (the Sepsis PREvalence Assessment Database, SPREAD): an observational study. Lancet Infect Dis..

[28] Rowan KM, Angus DC, Bailey M, Barnato AE, Bellomo R, PRISM Investigators (2017). Early, goal-directed therapy for septic shock—a patient-level meta-analysis. N Engl J Med..

[29] Righy C, do Brasil PEA, Vallés J, Bozza FA, Martin-Loeches I (2017). Systemic antibiotics for preventing ventilator-associated pneumonia in comatose patients: a systematic review and meta-analysis. Ann Intensive Care..

[30] Kaukonen K-M, Bailey M, Suzuki S, Pilcher D, Bellomo R (2014). Mortality related to severe sepsis and septic shock among critically ill patients in Australia and New Zealand, 2000–2012. JAMA.

